# Right top pulmonary vein is a venous anomaly of which surgeons should be aware in subcarinal dissection for thoracoscopic esophagectomy: a case report and literature review

**DOI:** 10.1186/s12957-022-02635-w

**Published:** 2022-05-20

**Authors:** Yuta Sato, Yoshihiro Tanaka, Shinya Ohno, Masahide Endo, Naoki Okumura, Nobuhisa Matsuhashi, Takao Takahashi, Takuya Saiki, Kazuhiro Yoshida

**Affiliations:** 1 Department of Gastroenterological Surgery and Pediatric Surgery, Gifu University Graduate School of Medicine, 1-1 Yanagido, Gifu City, Gifu Prefecture 501-1194 Japan; 2 Medical Education Development Center, Gifu University, 1-1 Yanagido, Gifu City, Gifu Prefecture 501-1194 Japan

**Keywords:** Esophageal cancer, Thoracoscopic esophagectomy, Right top pulmonary vein, Pulmonary vein, Navigation surgery

## Abstract

**Background:**

A right top pulmonary vein (RTPV) that crosses behind the right main or intermediate bronchus is a variation of the superior posterior pulmonary vein in the right upper lobe. Damage or ligation of this abnormal vessel can lead to massive intraoperative bleeding and serious complications, such as congestion of the posterior segment of the right upper lobe and cardiac tamponade. Subcarinal lymph node dissection is mandatory in radical thoracoscopic esophagectomy for esophageal cancer, and the RTPV is an anomalous vessel of which thoracic surgeons should be aware.

**Case presentation:**

A 70-year-old man underwent thoracoscopic esophagectomy for esophageal cancer (T3N1M0). An anomaly of the superior posterior pulmonary vein in the right lobe was recognized on preoperative computed tomography imaging. With simulation and intraoperative navigation using three-dimensional imaging of the same view as that observed during the operation, radical subcarinal dissection could be performed with preservation of the RTPV.

**Conclusion:**

In our review of the relevant literature, the incidence of RTPV ranged from 0.28 to 9.3%, and its mean vascular diameter was 7.0 mm at the maximum and 2.2 ± 0.72 mm at the minimum, with the right superior pulmonary vein being a relatively common inflow site. Our case in which the RTPV ran dorsal to the right main bronchus is very rare. In radical subcarinal dissection of thoracoscopic esophagectomy, it is important to recognize the posterior pericardial plane and release the ventral fixation of these lymph nodes to free space for the back side. This is also true in the case of RTPV, which should be noted to avoid injury. In cases involving an RTPV larger than 4.5 mm, ligation should be avoided, and preoperative recognition of the exact run of this abnormal vessel using three-dimensional imaging can be very useful.

## Background

Pulmonary veins have many anatomical variations, some of which may be affected during esophagectomy for esophageal cancer. The right posterior upper lobe segment vein, known as the right top pulmonary vein (RTPV), is rare anomalous vein running behind the right main or intermediate bronchus from the right upper lobe. The subcarinal portion of the RTPV penetrates the subcarinal lymph nodes (SCLNs). It is important to recognize this vein because SCNL dissection is an essential procedure for curative esophagectomy for esophageal cancer [1].

## Case presentation

**Fig. 1:**
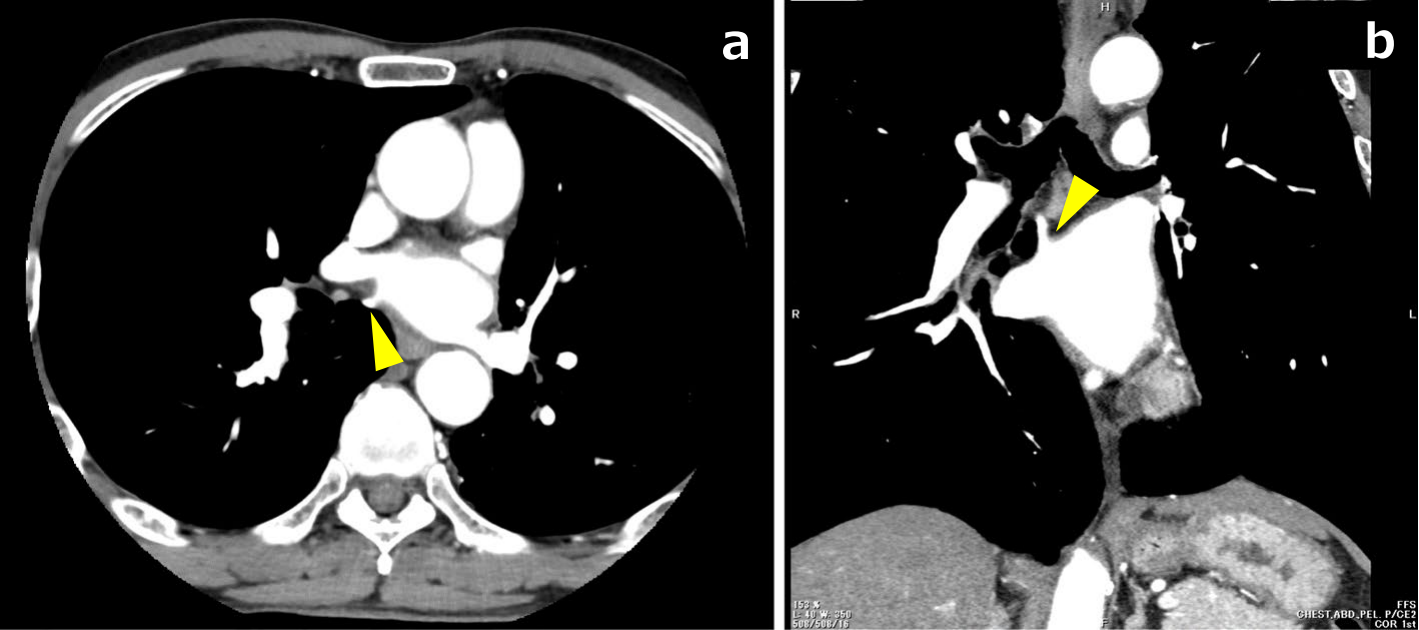
**a** Preoperative contrast-enhanced computed tomography showing the right top pulmonary vein dorsal to the right main bronchus (yellow arrowhead). **b** This abnormal vessel merges with the left atrium (yellow arrowhead)

**Fig. 2:**
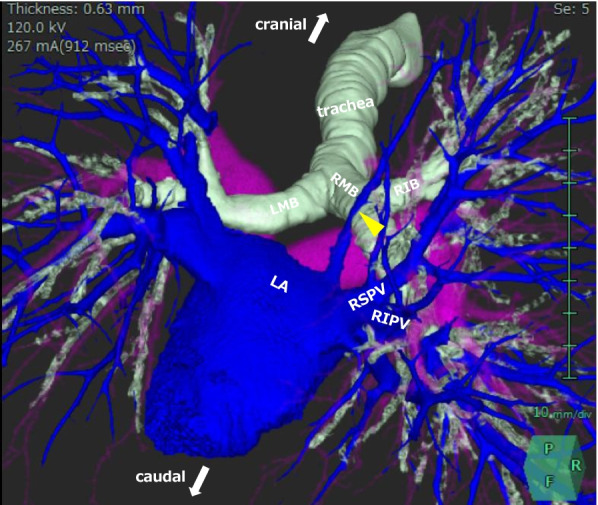
A three-dimensional image clearly demonstrates the right top pulmonary vein (yellow arrowhead) crossing behind the right main bronchus (RMB). RIB, right intermediate bronchus; LMB, left main bronchus; LA, left atrium; RSPV, right superior pulmonary vein; RIPV, right inferior pulmonary vein

A 70-year-old man complained of difficulty in swallowing food and was referred from another hospital. He was diagnosed with esophageal squamous cell carcinoma T3N1M0 in the median thoracic esophagus, according to the TNM classification, eighth edition. We therefore selected neoadjuvant chemotherapy (bi-weekly DCF: docetaxel 35 mg/m^2^, cisplatin 40 mg/m^2^, fluorouracil 400 mg/m^2^) as preoperative treatment [2, 3]. Preoperative contrast-enhanced computed tomography (CT) imaging revealed an aberrant branch of right posterior segmental vein (V^2^) passing behind the right main bronchus and merging with left atrium (LA) (Fig. 1). We used a high-speed three-dimensional (3D) image analysis system (SYNAPSE VINCENT, Fuji Photo Film Co., Ltd., Tokyo, Japan) to convert the Digital Imaging and Communication in Medicine (DICOM) data of contrast-enhanced CT images to 3D images, and the RTPV was clearly visualized as it appeared in the operative view in the supine position (Fig. 2). Surgery was performed 4 weeks after the end of neoadjuvant chemotherapy. Thoracoscopic esophagectomy with three-field lymphadenectomy via the right thoracic approach was conducted in the prone position using 6 ports. We synchronized the surgical image and the 3D image on the monitor side-by-side for virtual navigation and the precise location of the RTPV was recognized during surgery.

**Fig. 3:**
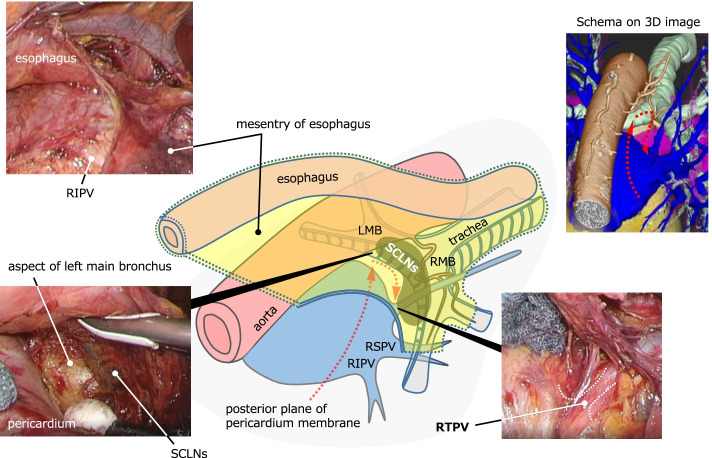
Intraoperative findings and schematic illustration. The first step in subcarinal lymph node (SCLN) dissection. Dissect the posterior plane of the pericardium membrane and identify the rise of the right top pulmonary vein (RTPV) after it reaches the aspect of the left main bronchus (red dot arrow in the schematic illustration)

**Fig. 4:**
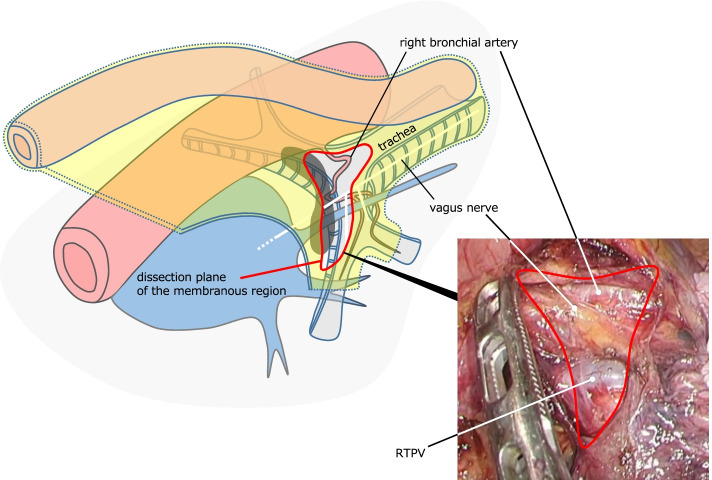
Intraoperative findings and schematic illustration of the second step in SCLN dissection. Expose the RTPV in the membranous region of the right main bronchus, preserving the right bronchial artery and the pulmonary branch of the vagus nerve (red dot line in the schematic illustration)

**Fig. 5:**
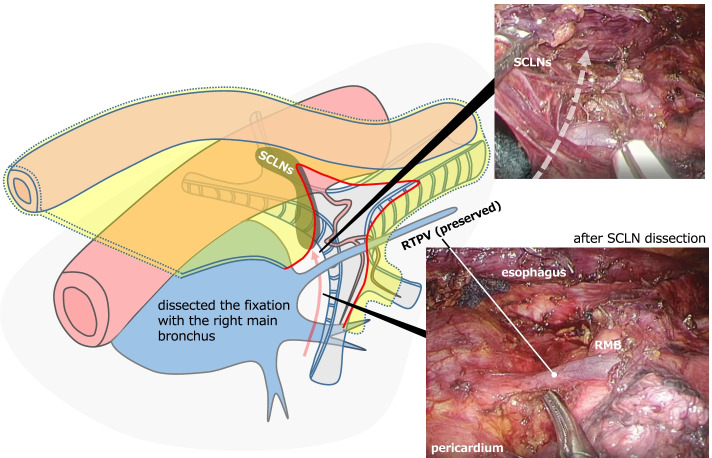
Intraoperative findings and schematic illustration of the final step of SCLN dissection. Complete the SCLN dissection by dissecting the fixation between the SCLN and the right main bronchus (red arrow in the schematic illustration)

SCLN dissection started from dissection of the posterior plane of the pericardium membrane. This plane continued to the anterior side of the SCLNs, reaching the cartilage of the tracheal bifurcation and the left main bronchus by recognizing the exact plane and expanding to the cranial and left side. Careful spreading of this released layer to the right revealed the rise of the RTPV embedded in the SCLNs (Fig. 3). Next, the dissection plane was switched to the membranous region of the right main bronchus, exposing the RTPV slightly from the distal side, while preserving the right bronchial artery and the pulmonary branch of the vagus nerve (Fig. 4). Finally, the fixation with the right main bronchus was dissected from the caudal side of the right main bronchus toward the direction of the inferior tracheal bifurcation to complete SCLN dissection for radical esophagectomy without damaging the RTPV (Fig. 5). After the thoracoscopic procedure, we reconstructed the gastric tube via a retrosternal route and hand-sewn anastomosis was performed at the left neck [4]. The operative time was 360 min, and the operative blood loss was approximately 30 mL. The patient recovered without complications and was discharged on the 21st day after surgery, although it took time for rehabilitation of muscle weakness.

## Discussion and conclusions

RTPVs are mainly reported by radiologists, cardiologists, and surgeons. Webb, a radiologist, first reported this anomalous vein using 2-dimensional CT [5]. Since atrial fibrillation is caused by electrical activity originating from the pulmonary veins, an accurate understanding of the pulmonary vein anatomy is important for catheter ablation therapy and has been reported from many Western countries [6–8]. In our review of the relevant literature, the incidence of RTPV ranged from 0.28 to 9.3%. Inflow sites included the right superior pulmonary vein (RSPV), the right inferior pulmonary vein (RIPV), the LA, and the V^6^, with the RSPV being relatively common and the V^6^ being very rare (Table 1). The location of the RTPV in relation to the right bronchus is mostly in the right intermediate bronchus. Cases such as ours, in which the RTPV runs dorsal to the right main bronchus, are very rare. Akiba et al. categorized the RTPV into six types based on their inflow site and route and reported that most types cross the intermediate bronchus and drain into the LA or pulmonary vein [9]. On the other hand, Miyamoto et al. classified the RTPV into four types based on the inflow site and reported that the type that drained into the inferior pulmonary vein was the most common [10]. Thoracic surgeons should be aware of these classifications and their frequency when performing radical esophagectomy, in which subcarinal lymph node dissection is mandatory.

**Table 1:** Review of previous literature about right top pulmonary vein and related case

			Occurent rate (%)			Location		Inflow site			
Author	Year	Specialty		*N*	Diameter (mm)	main	intermediate	SPV	IPV	LA	V^6^
Webb [5]	1984	Radiology	2.5	1/40		0	1	0	0	1	0
Jardin	1986	Radiology	9.3	10/107		0	10	0	10	0	0
Kim	1995	Radiology	5	14/280		0	14	3	11	0	0
Matsubara [11]	2003	Surgery	0.28	2/700		0	1				
Kato	2003	Cardiology	3.63	2/55				0	0	2	0
Lickfett [6]	2004	Cardiology	3.29	3/91	7.0	1^a^		0	0	3	0
Asai	2005	Surgery	5.7	41/725	4.1 ± 1.6	0	41	22	17	0	2
Weerasooriya [7]	2005	Cardiology	1.19	1/84				0	0	1	0
Kaseno	2008	Cardiology	3.73	16/428	5.6 ± 2.8			0	0	16	0
Arslan [12]	2008	Radiology	2.29	14/610	5.1	0	14				
Yamada [13]	2009	Surgery	1.16	1/86		0	1	0	0	1	0
Akiba	2010	Surgery	2.14	3/140		0	3	0	0	3	0
Akiba [9]	2013	Surgery	3.3	10/303		2^b^	9^b^	4	0	4	1
Shi [8]	2017	Radiology	0.98	1/102				0	0	1	0
Miyamoto [10]	2021	Surgery	8.01	31/387	2.2 ± 0.72	0	31	3	13	11	4
Yaginuma [14]	2021	Respiratory	3.29	154/4673	3.7			85	65	3	0
Our case		Surgery		1	5.8	1				1	

^a^The other two cases are not mentioned

^b^One example had two common branches

*RTPV* Right top pulmonary vein, *N* Number of patients, *SPV* Superior pulmonary vein, *IPV* Inferior pulmonary vein, *LA* Left atrium, *V*^*6*^ the branch of the right inferior pulmonary vein

Only 5 case reports in the literature have described the association of RTPV and SCLN dissection in esophagectomy [11, 15–18] (Table 2). All the cases, including ours, were reported from Japan, where radical esophagectomy with three-field lymphadenectomy is considered a standard procedure. The operative methods included open thoracotomy, thoracoscopy, and laparoscopic transhiatal approach, and—in all cases—the RTPV could be preserved during SCLN dissection. In thoracoscopic esophagectomy, it has been reported that the prone position is better for securing a good surgical field of view to identify the RTPV because the posterior upper lobe segment (S^2^) is located in the dorsal side of the right upper lobe [18]. It has been reported that retrosternal reconstruction may be a better method to avoid damaging the RTPV while pulling up the gastric tube [16]; we therefore chose this route. In addition, an increased incidence of incomplete fissure and displaced bronchus (DB) has been reported in patients with RTPV [14]. It is important to recognize the presence of the RTPV preoperatively because DB may lead to difficulty during differential lung ventilation and can lead to bronchial and vascular injury [19]. Preoperative simulation and intraoperative navigation with 3D images, which can be freely rotated and interactively visualized from any angle, are useful methods to enhance the surgeon’s understanding of the anatomy [20–22]. The use of 3D imaging enabled the preoperative diagnosis of three cases, including the present case.

**Table 2:** Review of literature on esophagectomy with the RTPV

Author	Year	Age	Sex	Location	Origin	Inflow site	Operative method	Position	Reconstruction route	Time (min)	Blood loss (ml)	Preoperative recognition	Preservation
Matsubara [11]	2003	57	M	Intermediate	Right upper lobe	LA	Thoracotomy	Decubitus				No	Yes
Fujiwara [15]	2015	51	M	Intermediate	S2	LA	Thoracoscopy	Decubitus	PMR			No	Yes
Shinozaki [16]	2016	74	M	Main	Right upper lobe	LA	Laparoscopy (transhiatal)	Supine	RR	378	169	No	Yes
Onodera [17]	2019	61	M	Intermediate	S2	RSPV	Thoracoscopy	Prone	PMR	815	52	Yes	Yes
Matubara [18]	2020	77	M	Intermediate	S2	RSPV	Thoracoscopy	Prone	PMR	620	150	Yes	Yes
Our case		70	M	Main	S2	LA	Thoracoscopy	Prone	RR	360	30	Yes	Yes

*RTPV* Right top pulmonary vein, *LA* Left atrium, *RSPV* Right superior pulmonary vein, *PMR* Posterior mediastinum rout, *RR* Retrosternal rout

An important step in SCLN dissection in thoracoscopic esophagectomy is to recognize the posterior plane of the pericardium and release the ventral fixation of the SCLNs to the free space at the back side. This procedure is important, even in the RTPV cases. After confirming the rise of the RTPV, careful encirclement of the RTPV then results in mesenterization of the SCLN, leaving only its fixation to the right main bronchus.

In our review of the relevant literature, the mean vascular diameter was 7.0 mm at the maximum and 2.2 ± 0.72 mm at the minimum (Table 1). The diameter of the RTPV may be correlated with the amount of venous blood flow from the right upper lobe (S^2^) [12]. If the RTPV is injured, hemostasis is required in the narrow surgical field due to the massive blood flow from the LA or pulmonary vein [11] and may cause cardiac tamponade [15]. It is also reported that if the large RTPV is ligated, the RTPV should be reconstructed or the S^2^ should be resected because it is considered to drain the entire venous flow from the S^2^, which may cause congestion of the S^2^ [15]. On the other hand, a case has been reported in which the RTPV was ligated and cut during right superior segmentectomy [13]. Although the article did not describe the diameter of the RTPV, no serious complications occurred. It was also reported that no symptoms suggestive of upper lobe congestion occurred after ligation if the RTPV was 4.5 mm or smaller [10]. Based on our review of the relevant literature, an RTPV larger than 4.5 mm should be noted in order to prevent injury and ligation should be avoided. The preoperative recognition of this abnormal vessel using 3D imaging was very useful for radical SCLN dissection during thoracoscopic esophagectomy.

## Data Availability

Not applicable.

## Abbreviations

RTPV: Right top pulmonary vein
SCLN: Subcarinal lymph nodes
CT: Computed tomography
V^2^: Branch of right posterior segmental vein
LA: Left atrium
3D: Three-dimensional
DICOM: Digital Imaging and Communication in Medicine
RSPV: Right superior pulmonary vein
RIPV: Right inferior pulmonary vein
S^2^: Posterior upper lobe segment
DB: Displaced bronchus
